# Dr. Egbert Le Fevre

**Published:** 1914-05

**Authors:** 


### OBITUARY

Readers are requested to report promptly the death of all physicians in Western New York, or former residents of this region, or graduates of any medical school in Western New York, and to notify the families of the deceased of our desire to publish adequate obituary notices.

**Dr. Egbert Le Fevre,** N. Y. Medical College, 1884, died suddenly of anginose scarlet fever, March 30, aged 56. He was the Dean of the Bellevue Hospital Medical College, author of a well known work on Physical Diagnosis, and a man esteemed both as a physician and for his personal qualities.

